# Knockdown of ST6Gal-I increases cisplatin sensitivity in cervical cancer cells

**DOI:** 10.1186/s12885-016-2981-y

**Published:** 2016-12-16

**Authors:** Xiaopeng Zhang, Chunchen Pan, Lei Zhou, Zhaogen Cai, Shufang Zhao, Donghong Yu

**Affiliations:** 1 Department of Obstetrics and Gynecology, The First Affiliated Hospital of Anhui Medical University, Hefei, 23022 China; 2 Department of Otolaryngology-Head and Neck Surgery, Anhui Provincial Hospital, Anhui Medical University, Hefei, 23000 China; 3 Department of Pathology, First Affiliated Hospital, Bengbu Medical College, 287 Changhuai Road, Bengbu, 233000 China

**Keywords:** α-2,6-sialic acid transferase, DDP, HeLa, Apoptosis, Invasion

## Abstract

**Background:**

Sialyltransferase I (ST6Gal-I) is an enzyme involved in tumor metastasis that processes sialic acid precursors into their mature form, enabling them to regulate gene expression. However, the effect of ST6Gal-I on the biological behavior of cancer cells remain unclear. This study was the first to demonstrate the influence of ST6Gal-I on cisplatin sensitivity in cervical cancer cells.

**Methods:**

Knockdown of ST6Gal-I was performed by shRNA and HeLa cells combination with cisplatin were tested.

**Results:**

We showed that down-regulation of ST6Gal-I promoted cell apoptosis and inhibited proliferation and invasion in cervical cancer cells. Knockdown of ST6Gal-I by RNA interference increased the sensitivity of HeLa cells to cisplatin in vitro, and reduced tumor volume and suppressed subcutaneous tumor growth in response to cisplatin treatment in a xenograft mouse model in vivo.

**Conclusions:**

The results provide new information that ST6Gal-I plays an important role in several biological or pathological processes including drug resistance in cervical cancer and may be a potential therapeutic target to improve the response to chemotherapy in cervical cancer patients.

**Electronic supplementary material:**

The online version of this article (doi:10.1186/s12885-016-2981-y) contains supplementary material, which is available to authorized users.

## Background

Cervical cancer is the second largest class of malignant tumors for women, and it endangers women's health, especially in developing countries [1]. On a global scale, approximately 500,000 new cases of cervical cancer are reported annually and approximately 230,000 women die of cervical cancer each year [2]. According to statistical data from the International Agency for Research on Cancer (IARC), in 2012 cervical cancer was the fourth most prevalent type of malignancy (62,000 new cases and 30,000 deaths) in Chinese women [3]. Although the prevalence is moderate compared with other regions, the mortality rate remains high, especially in rural areas. In addition, most cases occur at 40-54 years of age, which could lead to enormous social devastation [3]. The traditional treatment of cervical cancer is surgery or radiation therapy [4]. Despite significant improvements in surgical techniques and radiotherapy for the treatment of cervical cancer, its overall survival rate remains low. Research into the development and progression of this disease has shown that cervical cancer is a tumor that is sensitive to chemotherapy [5]. New treatment strategies including neoadjuvant chemotherapy (NAC) have been developed, and chemotherapy administered prior to the treatment of cancer can be differentiated from the second-line treatment following surgery [6]. However, metastasis and invasion are the main causes of death in cervical cancer patients, underscoring the importance of elucidating the molecular mechanisms underlying the progression of this disease [1]. Cytotoxic drugs such as cisplatin (DDP) can activate DNA damage signaling pathways [7, 8]. DDP-based regimens are frequently associated with severe side effects, including myelosuppression, asthenia and gastrointestinal disorders, as well as long-term cardiac, renal and neurological consequences, which are a frequent cause of poor tolerability, limited therapeutic efficacy, and drug discontinuation [9]. A major clinical obstacle in cancer therapy is the development of resistance to a multitude of chemotherapeutic agents, a phenomenon called multidrug resistance (MDR) [10]. Therefore, the design of new therapies capable of reversing chemotherapy resistance and enhancing sensitivity to platinum-based chemotherapy drugs is critical [11].

The extracellular matrix (ECM) is an important regulator of cell behavior and the microenvironment. The components of the ECM include fibronectin (Fn), collagen (Col), laminin (Ln), proteoglycans and non-matrix proteins [12]. Enhanced tumor cell adhesion to the ECM is a key step of cell invasion in tumor metastasis [13]. Integrins are transmembrane glycoproteins that form non-covalent heterodimers composed of α- and β-subunits. Members of the integrin family are the major cell surface receptors for the ECM and play a crucial role in mediating cell-ECM interactions during cell proliferation and tumor development, in addition to their involvement in the malignant behavior of tumors [14]. Glycosylation is a tissue-specific post-translational modification that is developmentally regulated by the activity of glycosyltransferases and glycosidases [15]. Although integrin-dependent cell adhesion is based on the binding of integrin to specific sequences in ECM proteins, this interaction is regulated by various factors including glycosylation modification [16]. The synthesis of α 2,6-linked sialic acid is catalyzed by β-galactoside: α 2,6-sialyltransferase 1 (ST6Gal-I), which adds sialic acid attached to Galβ1-4GlcNAc in an α2,6 linkage.

Elevated levels of ST6Gal-I and α2,6-linked sialic acid have been observed in carcinomas of the cervix, brain and liver [17–19]. In particular, the expression of the sialyltransferases, a family of anabolic enzymes that transfer sialic acid from CMP-NeuAc^2^ to glycoproteins or glycolipids, is altered in carcinoma cells of different origin. For instance, the activity of human Galβ1-4GlcNAc ST6Gal-I is low or not present in normal colonic mucosal cells but high in metastasizing colorectal carcinomas. In human breast carcinomas, high ST6Gal-I expression is associated with poor prognosis [20]. However, the role of ST6Gal-I in cisplatin chemo-resistance in cervical cancer is unknown.

RNA interference (RNAi) has been used to modulate gene expression in research laboratories around the world. It is a powerful genetic tool in biology and medicine for the elucidation of molecular pathways in organismal development and human disease [21]. Our previous collaborators had performed similar experiments with an additional breast carcinoma MDA-MB-435 cell line to validate the effect of ST6Gal-I [20]. However, there was no report about the effect of ST6Gal-I in cervical cancer cells. On the basis of these, we are the first and yet, mainly to demonstrate the influence of experimentally induced alterations in ST6Gal-I expression on cisplatin sensitivity in cervical cancer cells.

We showed that siRNA mediated knockdown of ST6Gal-I in HeLa cells down-regulated cell surface α2,6-linked sialic acid. We examined the effect of ST6Gal-I down-regulation on the response to cisplatin by assessing apoptosis and the invasive ability of cervical cancer cells in vitro and in human xenografts in nude mice. Our results suggest a potential novel therapeutic strategy for DDP-resistant cervical cancer and provide evidence of its clinical efficacy and its effect on the reversal of drug resistance.

## Methods

### Cell lines and culture conditions

The cervical cancer cell line HeLa was purchased from the China Center for Type Culture Collection (CCTCC; Shanghai, China) and cultured in Dulbecco's Modified Eagle's Medium (DMEM)-low sugar (Gibco, Carlsbad, CA, USA) supplemented with 10% fetal bovine serum (FBS; Gibco, Carlsbad, CA, USA), 100 U/ml penicillin, and 100 μg/ml streptomycin (Gibco BRL,Grand Island,NY, USA) at 37 °C in a humidified atmosphere of 5% CO_2_. HeLa cells were passaged every 2–3 days using 0.25% trypsin (Gibco BRL,Grand Island,NY, USA) and 0.02% EDTA (Sigma Aldrich, USA).

### Effect of cisplatin on HeLa cells viability

A MTT Cell Proliferation and Cytotoxicity Detection Kit was used to measure cell viability. Briefly, HeLa cells (2 × 10^4^/ml) in the logarithmic phase were seeded in 96-well culture plates and cultured at 37 °C under a 5% CO_2_ atmosphere for 24 h. The culture medium was removed after the cells adhered to the plate wall. The cells were then incubated in 200 μl of medium with cisplatin (Sigma Aldrich, USA) (0,0.5,1,2,5,10 and 20 μmol/L). The blank control group was generated using an equal volume of culture medium without the drug. Each group consisted of six parallel wells, and each experiment was repeated three times for each group. The cells were cultured for predetermined times (24, 48, 72 and 96 h). Then, the culture medium was removed. The cells were treated with 20 μl of 3-(4,5-dimethylthiazol-2-yl)-2-5-diphenyl tetrazolium bromide (MTT) (5 mg/ml) for 4 h, and dissolved in 150 μl of dimethyl sulfoxide solvent reagent for 10 min on a trace oscillator. Absorbance (A_490_) was measured at 490 nm on an enzyme-linked immunosorbent assay plate reader. The inhibition rate was calculated using the following formula: cell proliferation inhibition rate = (the average of A_490_ values from the control group - the average of A_490_ values from the experimental group)/the average of A_490_ values from the control group × 100% [22]. All experiments were performed in triplicate and more than three wells were used for each treatment. A time-concentration curve was constructed using the average value from three tests. The drug concentration resulting in 50% inhibition rate (IC_50_) was calculated using the weighted linear regression method with GraphPad 6.0 software.

### Stable ST6Gal-I short hairpin RNA (shRNA) transfection

Short hairpin RNA targeting human ST6Gal-I (pGPU6/GFP/Neo-ST6Gal -I-hemo) and negative control shRNA (pGPU6/GFP/Neo-shNC) were designed and chemically synthesized by the Gene Pharmaceutical Technology Company (Gene Pharma, shanghai, China) as shown in Table 1. HeLa cells were seeded at a density of 3 × 10^5^ in six-well culture plates one day before transfection, and then were transfected transiently using Lipofectamine 2000 (Invitrogen Life Technologies, USA) in antibiotic-free Opti-MEM culture medium according to the manufacturer's instruction. Cells were incubated at 37 °C in a 5% CO_2_ incubator for 6 h and were cultured in complete medium for 48 h continuously after washing with phosphate-buffered saline (PBS). A complete medium containing 800 μg/ml G418 was added, and stably expressed transfected cells were obtained by being screened for 2 weeks. Strains were acquired by expanded culture and stored in liquid nitrogen prior to further analysis [22].

**Table 1:** shRNA sequences for ST6Gal-I

	Sequence
ST6GalIshRNA	
Sense:	5′-CUCUCAGUUGGUUACCACAdTdT-3′
Antisense:	5′-UGUGGUAACCAACUGAGAGdTdT-3′
Negative control shRNA	
Sense:	5′-UUCUCCGAACGUGUCACAUdTdT-3′
Antisense:	5′-AUGUGACACGUUCGGAGAAdTdT-3′

### Assessment of transfection efficiency by flow cytometry and western blotting

The HeLa transfectants growing exponentially as monolayers were detached with 0.25%trypsin/0.02%EDTA, washed with complete medium, and allowed to recover overnight in a 50 ml centrifuge tube. One half of the cells was treated with 100 mU *V. cholerae* sialidase in 0.1 M sodium acetate buffer pH 5.5 containing 9 mM CaCl_2_ and 154 mM NaCl for 1 h at 37 °C. The isotype control cells were incubated with buffer alone. Prior to the characterization of cell surface constituents, cells were washed with PBS and resuspended at a density of 1 × 10^6^ cells/ml in PBS. To assess cell surface 2,6-sialylation, FITC-labeled *S. nigraagglutinin* was used. Cells (1 × 10^6^) were suspended in 50 μl staining buffer (1% BSA in HBSS) containing 1 μg FITC-SNA and incubated for 1 h on ice. Flow cytometric analysis was carried out immediately after washing cells with HBSS.

Cells were harvested and homogenized in lysis buffer, followed by incubation on ice for 30 min. The homogenates were ultrasonicated, followed by centrifugation (Eppendorf model 5417R, Eppendorf, Hamburg) at 12000 revolutions/min for 30 min at 4 °C. Samples with equal protein (50 μg) were loaded on polyacrylamide gel and separated by electrophoresis at 90 V. Proteins were then transferred onto immobilon polyvinyldifluoride membranes (Millipore, MA, USA). Nonspecific binding was blocked in Tris-buffered saline + 0.2% Tween-20 containing 5% Bovine Serum Albumin for 2 h at room temperature. The membranes were incubated with primary antibody against ST6Gal-I (1:500; Santa Cruz Biotechnology,Santa Cruz,CA) overnight at 4 °C. The membranes were then incubated with secondary horseradish peroxidase conjugated goat anti-rabbit antibody (1:1,000). Protein bands were visualized with enhanced chemiluminescence reagents (Amersham Biosci., Piscataway, NJ, USA) and UVP imaging system (EC3-Imaging-System, Upland, CA, USA). Imaging signals were digitized and analyzed. The ratio of band intensity to β-actin was obtained for analysis.

### Annexin V-PI apoptosis assays

Cells were incubated and harvested after a 48 h treatment as described above. For Annexin V-propidium iodide (PI) assays, cells were stained and evaluated for apoptosis by flow cytometry according to the manufacturer's protocol. Briefly, 1 × 10^6^ cells were stained with 5 μl Annexin V-fluorescein isothiocyanate (FITC) and 10 μl PI (5 μg/ml) in 1 × binding buffer (1.0 mmol/L HEPES [4-(2-hydroxyethyl)-1-piperazineet

-hanesulfonic acid] pH = 7.4, 140 mmol/L NaOH, 2.5 mmol/L CaCl_2_) for 20 min at room temperature in the dark. Apoptotic cells were determined by flow cytometry (FACS Calibur,Becton-Dickinson, USA) using Cell Quest software (BD Biosciences, San Jose, CA, USA).

### TUNEL apoptosis assays

The TUNEL reaction was performed using the one step TUNEL apoptosis assay kit-green fluorescein (Beyotime Institute of Biotechnology, hangzhou, China) according to the manufacturer's instructions. Briefly, cells were fixed in 4% paraformaldehyde for 20 min. Cells were then incubated in immune dyeing washing liquid (0.1% Triton X-100 in PBS) for 2 min on ice before labeling with 50 μl TUNEL reaction mixture and incubating at 37 °C for 1 h in the dark. After washing, slides were mounted and examined in 10 randomly selected low-power fields (×200) using a fluorescence microscope. The percentage of apoptotic cells was calculated as (TUNEL-positive cells/total cells) × 100% [23]. All assays were performed in triplicate.

### Cell invasion assays

A Matrigel-based transwell assay was performed to determine the invasive properties of cells. Cells (1 × 10^5^/well) were trypsinized, resuspended in serum-free DMEM-low sugar medium and then added to the transwell inserts (6.5 mm diameter, 8 μm pore size, polycarbonate membrane; Corning Costar, Cambridge, MA, USA). DMEM-low sugar medium (500 μl) with 10% FBS was added to the lower chamber under the insert membrane and the transwell chambers were incubated for 24 h under culture conditions. The inserts were then washed with PBS, migrated cells on the lower surface of the membrane were fixed with 4% paraformaldehyde for 20 min, stained with hematoxylin-eosin (HE), and counted in 10 randomly selected low-power fields (×100) under a microscope. The average value was used as the parameter to evaluate the invasive ability of the cells. All assays were performed in triplicate.

### Tumor model and treatment

The animal study was approved by the Institutional Animal Care and Use Committee of Bengbu Medical College and in accordance with the Guide for the Care and Use of Laboratory Animals (NIH Publication No. 85-23, 1996). BALB/c nude mice (aged 4 weeks,female,mean body weight 20 g) were purchased from the Shanghai National Center for Laboratory Animals and kept in a specific pathogen-free environment where temperature was maintained at 22 °C and humidity in the range of 40–50%. To assess the effect on tumorigenicity, stably transfected ST6Gal-I-shRNA and NC-shRNA HeLa cells were harvested and the BALB/c nude mice were randomly subcutaneously inoculated in the right oxter with 1 × 10^7^ cells suspended in 0.2 ml of DMEM-low sugar medium to generate cervical cancer xenograft tumors. When tumors in the nude mice had reached an approximate volume of 200 mm^3^, as calculated by the following equation: tumor volume V (mm^3^) = 1/2 × a × b^2^ where a is the longest diameter and b is the shortest diameter, the tumor-bearing mice inoculated with ST6Gal-I-shRNA HeLa cells were randomly allocated to a DDP group and a control group (n = 6 for each group). Each mouse received an intraperitoneal injection of normal saline (0.2 ml) or DDP (7.5 mg/kg in 0.2 ml of normal saline). The tumor-bearing mice inoculated with NC-shRNA HeLa cells also received an intraperitoneal injection of DDP. After four weeks, the mice were sacrificed and tumors were harvested and processed for immunohistochemistry analysis to determine protein expression.

### Immunohistochemistry

Immunohistochemistry was performed on 4 μm sections of paraffin-embedded tissue blocks obtained from xenograft mice using the avidin-biotin immunoperoxidase method. The paraffin sections were deparaffinized with xylene and rehydrated in a decreasing ethanol series. Endogenous peroxidase activity was blocked by incubation for 10 min in 3% H_2_O_2_ buffer. To unmask the ST6Gal-I and BCL-2 epitopes, microwave processing pretreatment was carried out in citrate buffer, pH = 6.0 for 10 min. Sections from paraffin-embedded tumors were incubated overnight at 4 °C with rabbit anti-human ST6Gal-I polyclonal antibody (Santa Cruz Biotechnology,Santa Cruz,CA, USA) at 1:200 dilution and mouse anti-human BCL-2 monoclonal antibody (Santa Cruz Biotechnology,Santa Cruz,CA, USA) at 1:500 dilution, followed by incubation at 37 °C for 30 min with goat anti-rabbit or goat anti-mouse secondary antibody (Pierce, Rockford, IL, USA). Finally, slides were counterstained with hematoxylin, dehydrated in an ascending ethanol series, cleared with xylene, and mounted with coverslips using a permanent mounting medium. Immunohistochemical evaluation was performed independently by two senior pathologists who were blind to the section data. Ten high-power (×400) visual fields were randomly selected from each slide. Cytoplasmic staining which were presented as brown granular materials was considered to be positive for ST6Gal-I and BCL-2. The evaluation was analyzed semi-quantitatively according to both the percentage of positive cells and the intensity of staining. The intensity of positive staining was scored on a scale of 0 to 3, 0 records as negative, 1 as weak staining, 2 as moderate staining, 3 as strong staining. The percentage of positive cells was also scored on a scale of 0 to 4, no positive cells records as 0, <25% as 1, 26%–50% as 2, 51%–75% as 3, >75% as 4. The final score was determined by adding the intensity of positive staining and the percentage of positive cells, yielding a range from 0 to 7. The expression of ST6Gal-I and BCL-2 was considered positive when the scores were ≥2; 2 to 3 was considered weak; 4 to 5 was considered moderate; and 6 to 7 was considered strong [22, 24].

### Statistical analysis

Data were expressed as mean ± standard deviation (SD). The statistical significance of differences was estimated by a two-tailed Student's *t*-test or a two-way analysis of variance (ANOVA), as appropriate, using the Statistical Package for the Social Sciences (SPSS) 19.0 software. *P* < 0.05 was considered statistically significant.

## Results

### Effect of DDP on HeLa cells viability

**Fig. 1:**
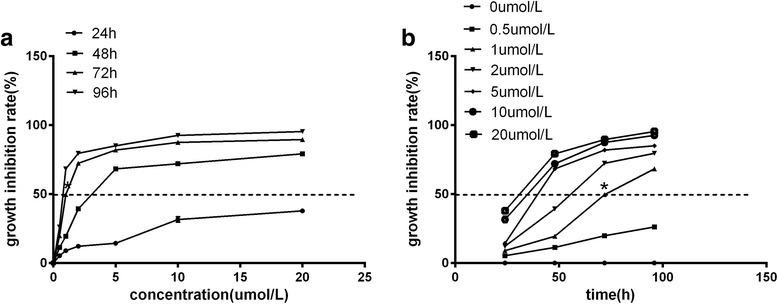
Effect of DDP on the HeLa cells growth inhibition rate at the indicated concentration (﻿**a**﻿) and time (﻿**b**﻿). *, *P* < 0.05; *N* = 3

DDP inhibited HeLa cells proliferation in a dose-dependent manner starting at a concentration of 0.5 μmol/L. At concentrations above 1 μmol/L, the inhibitory effect of DDP on HeLa cells proliferation was markedly increased. Significant differences in the inhibition of cells proliferation were observed between the control group and the DDP treatment groups, as well as among the different DDP treatment groups (*P* < 0.05). DDP inhibited proliferation in a time-dependent manner (*P* < 0.05, Table 2, Fig. 1). Based on the findings of our MTT assay, a DDP dose of 1 μmol/L and 72 h were selected as optimal conditions for targeted gene delivery.

**Table 2:** Inhibition of HeLa cell proliferation by DDP

DDP concentration (μmol/L)	I (24 h)	II (48 h)	III (72 h)	IV (96 h)
0	0	0	0	0
0.5^a^	5.27 ± 0.41	11.30 ± 1.02^f^	19.79 ± 0.92^g^	26.19 ± 0.63^h^
1^b^	8.94 ± 0.76	19.39 ± 0.88^f^	49.57 ± 1.08^g^	68.26 ± 1.31^h^
2^c^	12.17 ± 0.28	39.24 ± 0.72^f^	72.32 ± 0.67^g^	79.55 ± 0.22
5^d^	14.33 ± 0.69	68.26 ± 1.00^f^	81.95 ± 1.34^g^	85.03 ± 1.11
10^e^	31.57 ± 2.01	71.93 ± 0.33^f^	87.46 ± 0.86^g^	92.54 ± 0.92
20	37.85 ± 0.78	79.19 ± 1.12^f^	89.50 ± 1.13^g^	95.36 ± 0.48
IC50	46.30 ± 0.27	3.68 ± 0.16	1.24 ± 0.89	0.76 ± 0.05

^a^ *P* < 0.05;^b^ *P* < 0.05;^c^ *P* < 0.05;^d^ *P* < 0.05;^e^ *P* < 0.05; ^f^ *P* < 0.05;^g^ *P* < 0.05; ^h^ *P* <0.05

### Knockdown of ST6Gal-I by shRNA

**Fig. 2:**
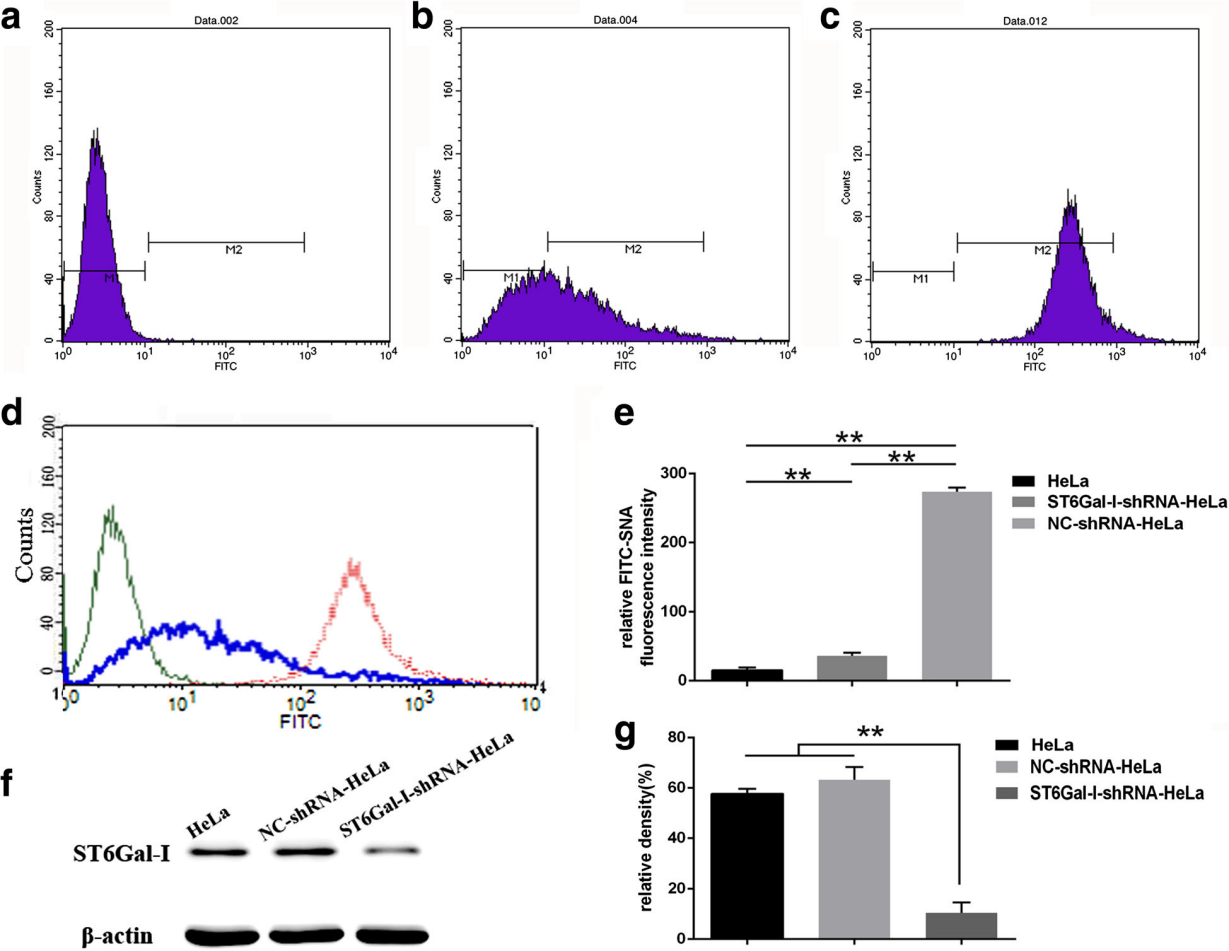
Stable transfection of cervical cancer cells using ST6Gal-I shRNA. Flow cytometry analysis of NC-shRNA and ST6Gal-I-shRNA cells with FITC-conjugated SNA lectin. (**a**) the isotype control group, (**b**) the ST6Gal-I-shRNA HeLa group, (**c**) the NC-shRNA HeLa group, (**d**) the three groups combination as a picture, (**e**) the relative fluorescence intensity was analysed; (**f**) the bands for three groups by western blotting, (**g**) the relative density for the bands was detected. Transfection efficiency was decreased in ST6Gal-I-shRNA HeLa cells group compared with NC-shRNA group and untransfected isotype control group. *, *P* < 0.05; *N* = 3

The function of ST6Gal-I in cervical cancer was examined by silencing ST6Gal-I in HeLa cells using ST6Gal-I-shRNA. Untransfected isotype HeLa cells and negative control siRNA (NC-shRNA)-transfected HeLa cells were used as controls. A 86.75% decrease in the level of FITC-SNA fluorescence intensity was observed in ST6Gal-I-knockdown HeLa cells compared with NC-shRNA-transfected HeLa cells by flow cytometry (*P* < 0.001, Fig. 2). Meanwhile analysis by western blotting further showed that ST6Gal-I protein was down-regulated significantly in the ST6Gal-I-shRNA HeLa group compared to the HeLa group (*P* < 0.05, Fig. 2f and g). The expression of ST6Gal-I protein was no significant difference between NC-shRNA HeLa group and HeLa group (*P* > 0.05, Fig. 2g). Downregulation of ST6Gal-I expression level after transfection was stable at least for 4 weeks (Additional file 1: Fig. S1).

### Down-regulation of ST6Gal-I increases apoptosis of cervical cancer cells

**Fig. 3:**
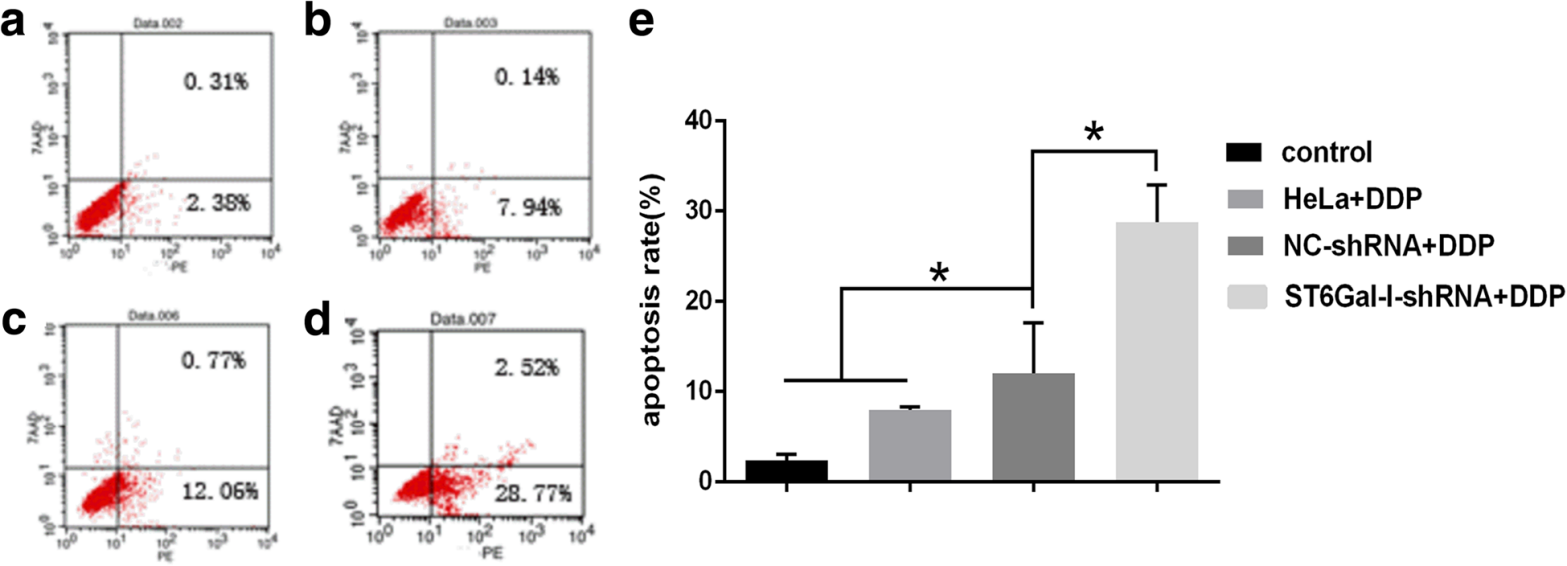
Apoptosis of HeLa cells treated with DDP for 48 h by flow cytometry. (**a**) the control group without DDP treatment, (**b**) the HeLa cells group, (**c**) the NC-shRNA cells group, (**d**) the ST6Gal-I-shRNA cells group, (﻿**e**﻿)﻿ The apoptosis rate of ST6Gal-I-shRNA cells was increased than that in other groups. *, *P* < 0.05; *N* = 3

Annexin V-PI double-staining assays showed that the rate of apoptosis in HeLa cells treated with DDP was 7.94% ± 0.36%, whereas that of the ST6Gal-I-shRNA + DDP group was 28.77% ± 4.11%, which was significantly higher than that of the NC-shRNA + DDP group (12.06% ± 5.55%) or the control group (2.38% ± 0.64%) (*P* < 0.05, all) (Fig. 3).

### TUNEL apoptosis assays

**Fig. 4:**
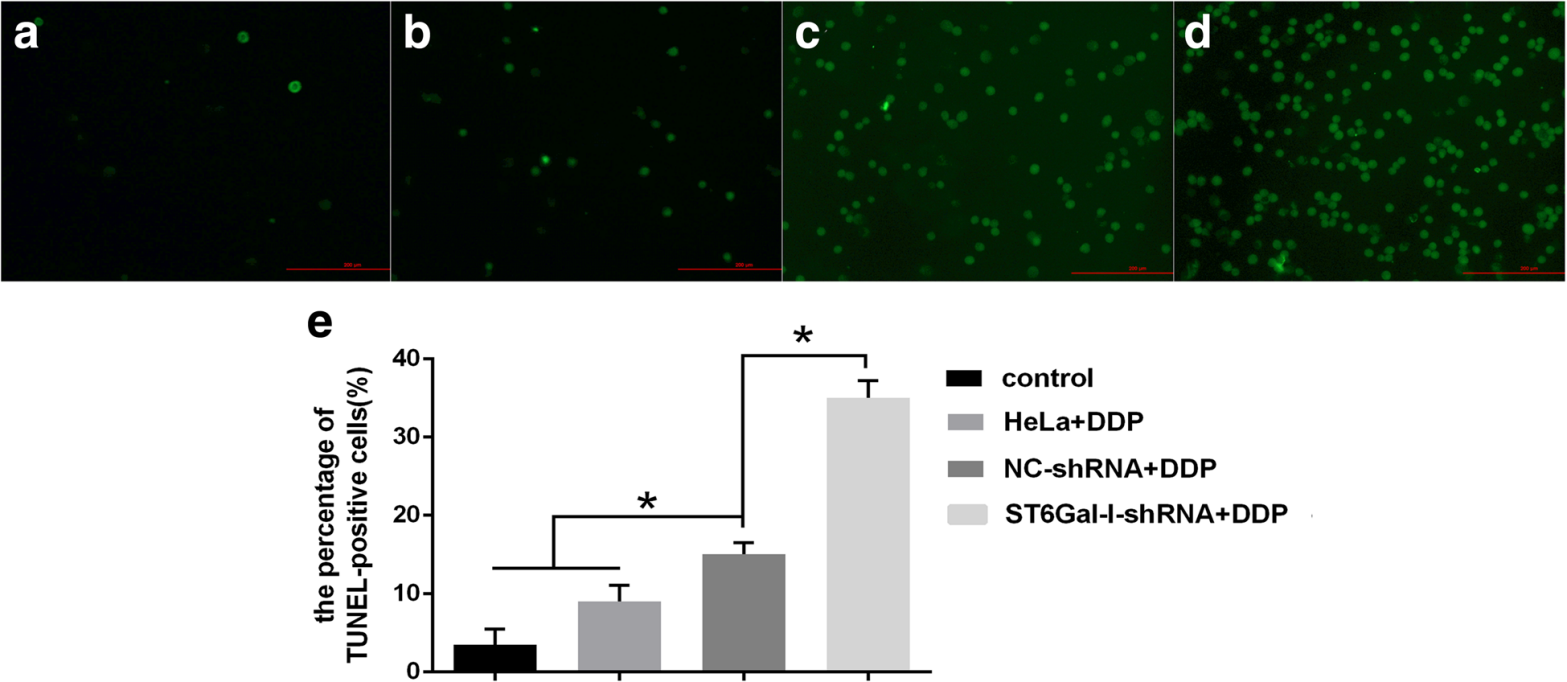
(**a**) representative fluorescence microscopic images of HeLa cells by TUNEL assays and (**b**) quantitative analysis of apoptosis. (**a**) the control group without DDP treatment, (**b**) the HeLa cells group, (**c**) the NC-shRNA cells group, (**d**) the ST6Gal-I-shRNA cells group, (**e**﻿) The up-regulation apoptosis rate in ST6Gal-I-shRNA cells group was further verified. *, *P* < 0.05; *N* = 3, scale bar 200 μm﻿

To confirm the above effect, HeLa cells were treated with DDP and examined by TUNEL assays. Treatment of HeLa cells and NC-shRNA HeLa cells with DDP had no significant effect, whereas DDP combined with ST6Gal-I-shRNA significantly increased the percentage of TUNEL-positive cells. These results were consistent with those obtained by flow cytometry and verified that the down-regulation of ST6Gal-I can increase the chemo-sensitivity of cervical cancer cells to cisplatin (Fig. 4).

### Down-regulation of ST6Gal-I inhibits the invasive ability of cervical cancer cells

**Fig. 5:**
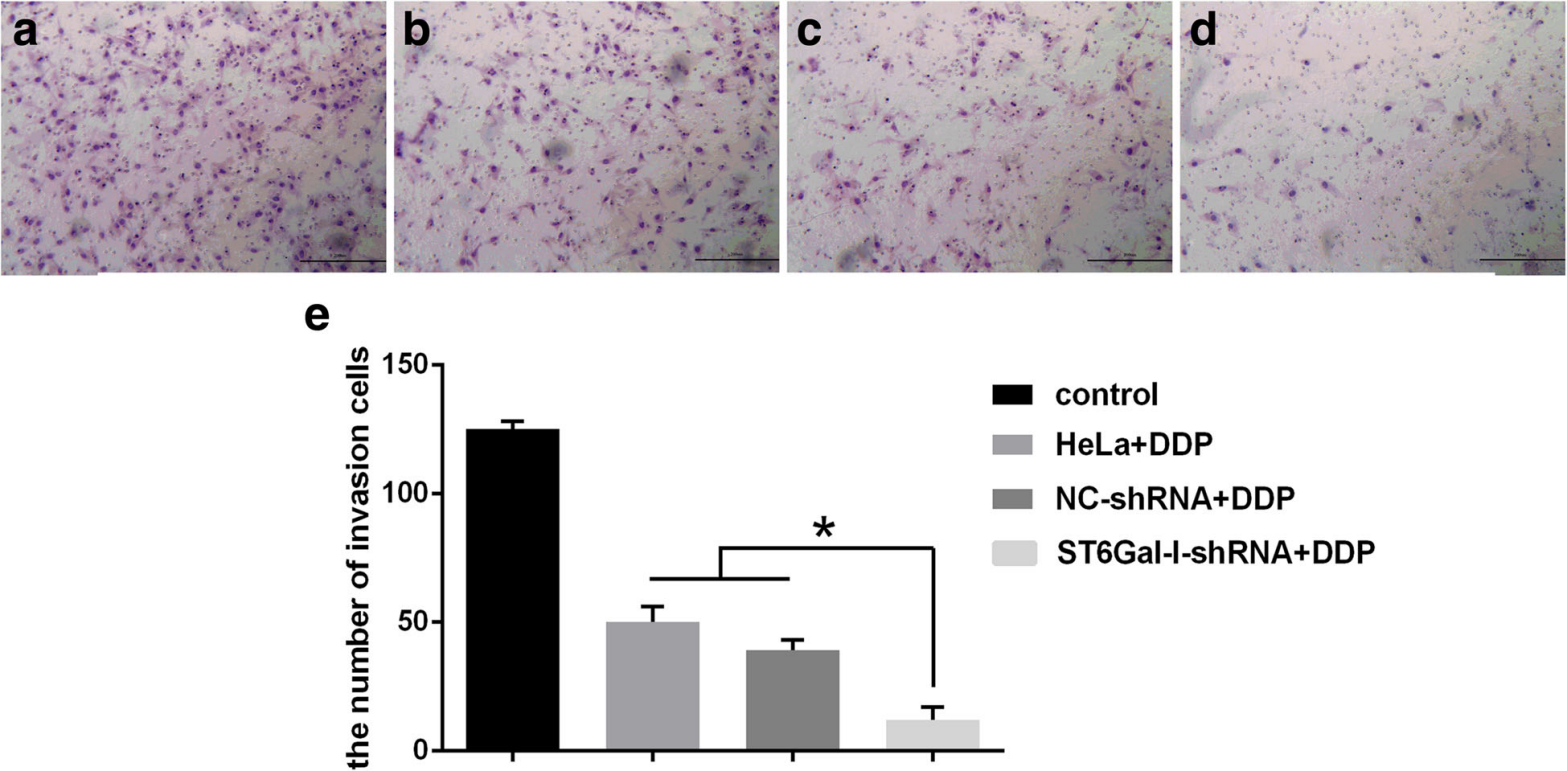
The invasion ability of HeLa cells by Transwell assays. (**a**) the control group without DDP treatment, (**b**) the HeLa cells group, (**c**) the NC-shRNA cells group, (**d**) the ST6Gal-I-shRNA cells group, (**﻿e**) The number of ST6Gal-I-shRNA cells passing through the membrane was significantly lower than that in other groups. *, *P* < 0.05; *N* = 3, scale bar 50 ﻿μm

To elucidate the effect of ST6Gal-I on invasion in HeLa cells in vitro, we performed transwell invasion assays in ST6Gal-I-shRNA transfected cells (Fig. 5). ST6Gal-I-shRNA transfected cells, NC-shRNA transfected cells and normal HeLa cells were seeded in the inserts. After 24 h, the number of cells migrating to the lower side of the 8 μm pore size membrane was calculated to quantify the invasive capacity. The number of cells that passed through the membrane was significantly lower in ST6Gal-I-shRNA cells than in the NC-shRNA transfected cells and normal HeLa cells (*P* < 0.05), suggesting that knockdown of ST6Gal-I decreases the invasive ability of HeLa cells in vitro.

### Inhibition of tumor growth

**Fig. 6:**
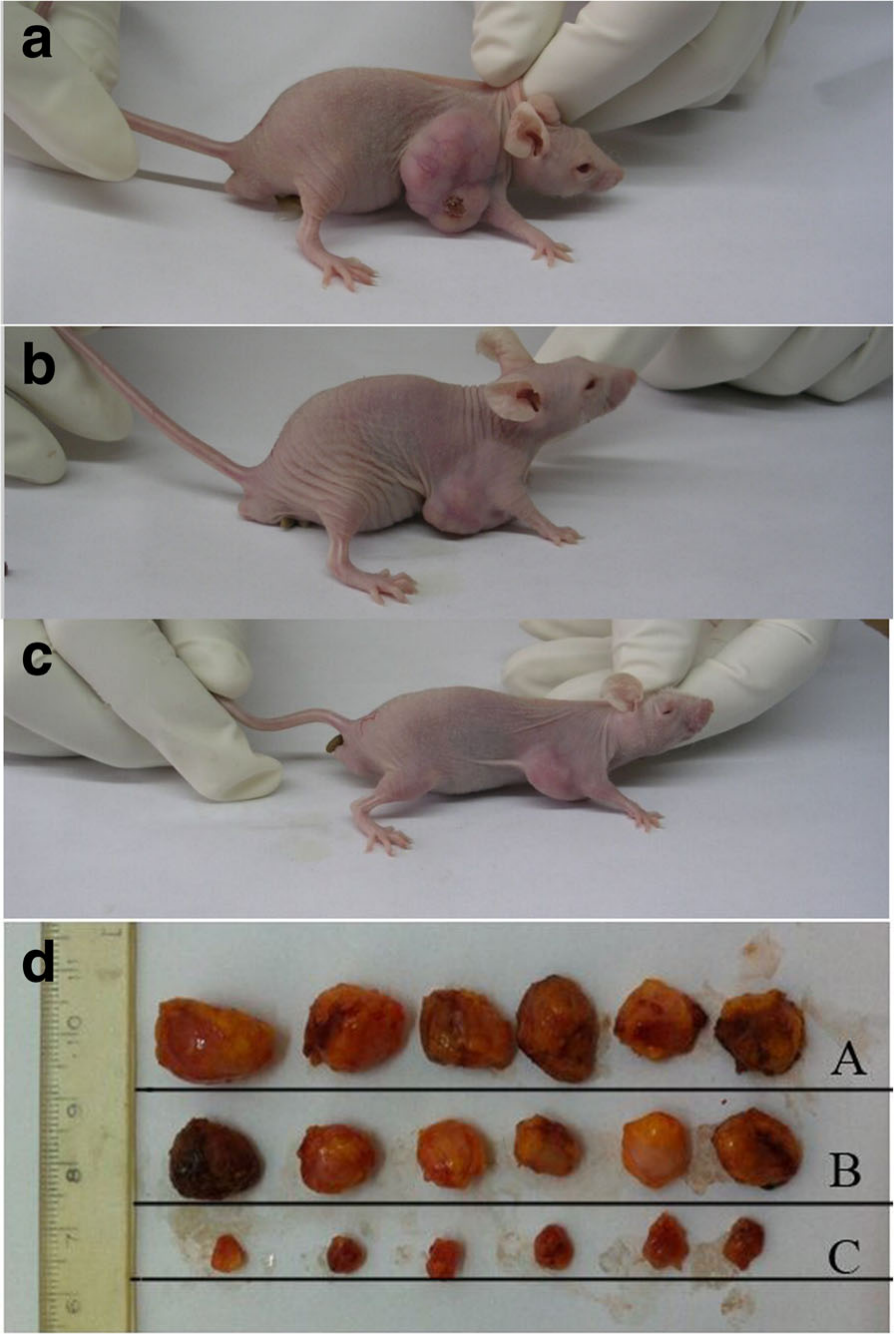
Tumor volume in nude mice with treated after four weeks. **a** the control group without DDP treatment, (**b**) the NC-shRNA + DDP group, (**c**) the ST6Gal-I-shRNA + DDP group. Tumor volume was smaller in the ST6Gal-I-shRNA + DDP group than that in other groups. ﻿**d﻿** Tumor isolated from nude mice with treated with the control group﻿ (A), the NC-shRNA+DDP group (B), and the ST6Gal-I-shRNA + DDP (C) group﻿﻿. ﻿*N* = 5

A preliminary in vivo study was performed to evaluate the antitumor activity of ST6Gal-I-shRNA. An appreciable lump was observed in the right oxter of each mouse approximately one week after injection of the transfected HeLa cells, and tumor volumes reached a mean of 200 mm^3^. Thereafter, the BALB/c nude mice were randomly assigned to the different treatment groups and treated for approximately four weeks. Tumor volume was higher in the control group (415 ± 38 mm^3^, Fig. 6a) than in the NC-shRNA + DDP group (285 ± 27 mm^3^, Fig. 6b), indicating moderate antitumor efficacy of DDP. Subcutaneous tumor growth was suppressed effectively in mice treated with ST6Gal-I-shRNA + DDP (149 ± 13 mm^3^, *P* < 0.05, Fig. 6c), suggesting that the presence of ST6Gal-I-shRNA enhanced the effect of DDP on the inhibition of tumor growth.

### Immunohistochemistry

**Fig. 7:**
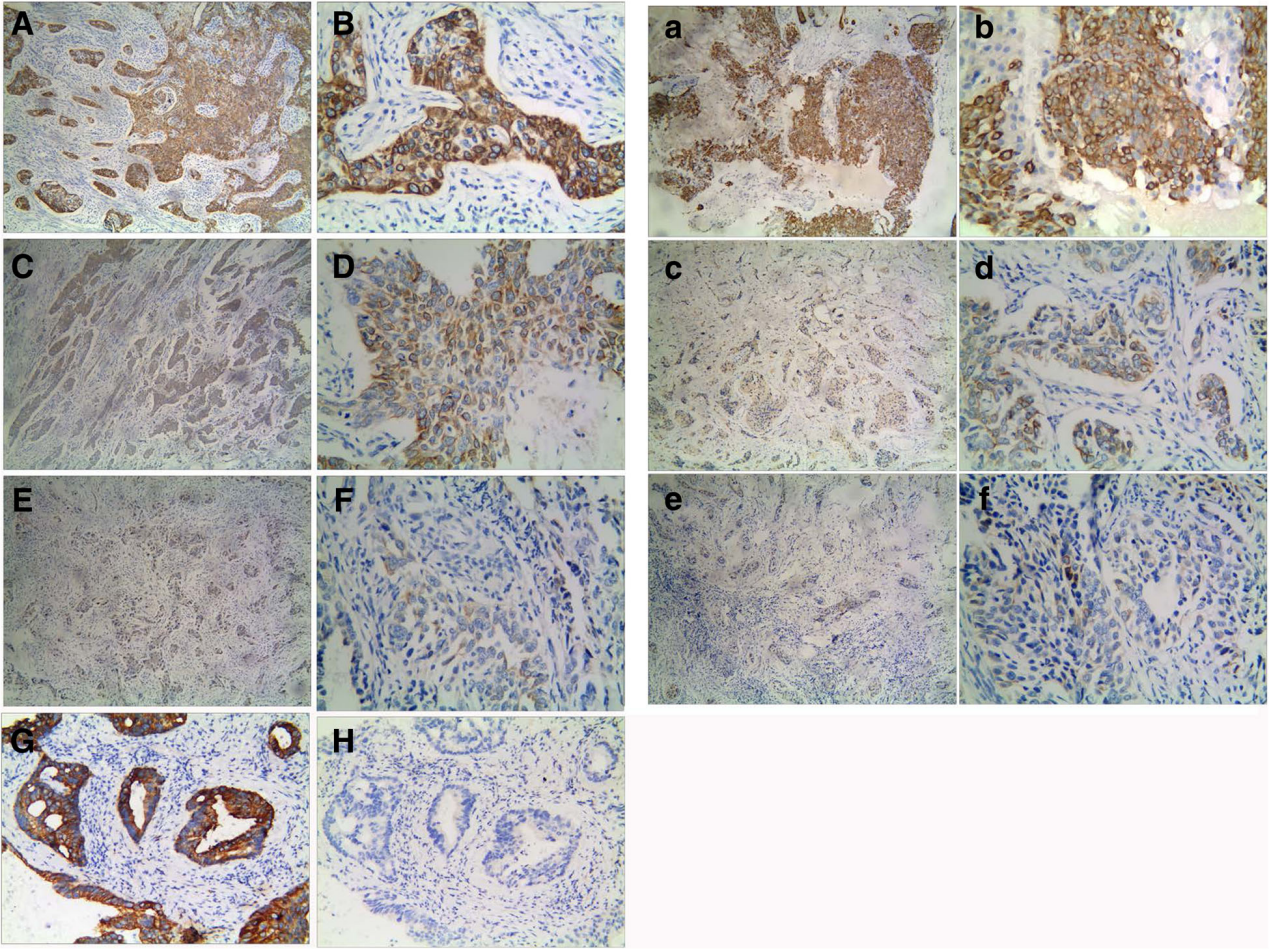
Immunohistochemistry staining for ST6Gal-I and BCL-2 protein in xenograft tumor model. Cytoplasmic staining was considered to be positive for ST6Gal-I and BCL-2. (**A**) + (**B**) matched samples with accentuated BCL-2 protein expression and (*a*) + (*b*) matched samples with strong positive ST6Gal-I expression for the control group (staining intensity 3). (**C**) + (**D**) matched samples with moderate BCL-2 protein expression and (*c*) + (*d*) matched samples with weaker positive ST6Gal-I expression for the NC-shRNA HeLa cells group (staining intensity 2). (**E**) + (**F**) matched samples with weakest positive BCL-2 expression and (*e*) + (*f*) matched samples with almost complete loss of ST6Gal-I protein for the ST6Gal-I-shRNA HeLa cells group (staining intensity 1). (**G**) Positive control: breast cancer sample with strong ST6Gal-I staining (staining intensity 3). (**H**) Negative control: breast cancer sample with no staining (staining intensity 0). **A**, **C**, **E**, **G**, **H**, *a*, *c* and *e* show 100 × amplification in the light microscope. **B**, **D**, **F**, *b*, *d*, and *f* show 400 × amplification in the light microscope

A preliminary in vivo study was performed to evaluate the antitumor activity of ST6Gal-I-shRNA. In order to further validate the effect of ST6Gal-I shRNA, we performed immunohistochemical ST6Gal-I protein expression in xenograft tumor tissues. ST6Gal-I protein staining was detected cytoplasmatically in HeLa cells, with strongest abundance in control groups (Fig. 7a and b). While tumors were characterized by a decreased ST6Gal-I protein staining treated with NC-shRNA + DDP (Fig. 7c and d). We found an almost complete loss of ST6Gal-I protein in the group treated with ST6Gal-I-shRNA + DDP (Fig. 7e and f). It showed that knockdown of ST6Gal-I indeed down-regulated the expression of ST6Gal-I in xenograft tumor model. At the same time, detection of BCL-2 protein expression in xenograft tumor tissues also showed the strongest positivity in the cytoplasm of tumor cells from the control groups (Fig. 7A and B), and a marked decrease in response to DDP treatment. Expression of BCL-2 was weaker in tissues treated with NC-shRNA + DDP (Fig. 7C and D), and the greatest effect was observed in the group treated with ST6Gal-I-shRNA + DDP (Fig. 7E and 7F), suggesting that the presence of ST6Gal-I-shRNA increased apoptosis effect in HeLa cells and enhanced the effect of DDP on the inhibition of tumor growth.

### Toxicity in mice

All of the mice tolerated the study agents satisfactorily, showing no gross signs of cumulative adverse effects such as weight loss, ruffling of fur, or behavioral and postural changes.

## Discussion

Platinum-based combination chemotherapy is the most widely used method in the treatment of cervical cancer, in particular in the late period and in patients showing relapse or metastasis. However, chemotherapy resistance frequently results in treatment failure. Therefore, reversing platinum resistance in cervical cancer and increasing sensitivity to platinum-based chemotherapy drugs are crucial issues. The molecular genetic basis of resistance to cancer therapeutics is complex, involving multiple processes such as drug transport, drug metabolism, DNA repair and apoptosis [25]. Furthermore, the factors regulating chemo-resistance in cervical cancer remain poorly understood.

Glycosylation, which is the most common posttranslational protein modification, can affect protein folding, stability, and function [26]. Numerous genes in the human genome encode glycan-synthesis-related proteins [27, 28] involved in generating the sugar chains that are linked to proteins to form glycoproteins; these sugars are classified as either N-linked glycan chains [29], which are bound to the amidic nitrogens of asparagines, or O-linked glycan chains [30], which are attached to the hydroxyl groups of serines or threonines. Cellular transformation is typically accompanied by alterations in the composition of glycoproteins, which are major constituents of the cell membrane, and abnormal glycosylation has been correlated with cancer progression and malignancy [31–34]. In particular, heavily sialylated N-glycans appear to be highly correlated with cancer metastasis [35–37]. In humans, N-acetylneuraminic acids (Neu5Ac) are the most prominent forms of sialic acid in N-glycan chains; these negatively charged monosaccharides are widely distributed as terminal sugars that coat the eukaryotic cell surface and participate in various biological processes, including cell-cell communication, inflammation, immune defense, and tumor metastasis [38]. Because of the important biological and cancer-related functions of these glycans, the biosynthesis, acceptor substrate transfer, degradation, and recycling of sialic acids have been studied extensively [39–41]. Glycosylation requires the coordinated activity of various glycosyltransferases that catalyze the transfer of monosaccharide residues from nucleotide sugar donors to specific acceptor substrates, forming glycosidic bonds. Sialyltransferases are key enzymes in the biosynthesis of sialic acid-containing glycoproteins and glycolipids. They constitute a subset of glycosyltransferases that use cytidine monophosphate (CMP) to catalyze the transfer of sialic acids to the ends of carbohydrate chains linked to glycoproteins or glycolipids [39, 42, 43]. Twenty members of the mammalian sialyltransferase family have been identified to date [44, 45]. They are divided into four subfamilies according to their synthesized carbohydrate linkages: β-galactoside α2, 3-sialyltransferases (ST3Gal I-VI); β-galactoside α2, 6-sialyltransferases (ST6Gal I and II); GalNAc α2, 6-sialyltransferases (ST6GalNAc I-VI); and α2, 8-sialyltransferases (ST8Gal I-VI). ST6Gal-I catalyzes the formation of α(2,6) linkages; however, it specifically targets terminal Galβ(1,4)GlcNAc structures in glycoproteins. One of the most significant glycosylation-related changes is the elevation of ST6Gal-I activity in tumor tissues compared with the surrounding healthy mucosa. Additionally, several clinical studies conducted over the past few years have shown that the activity of ST6Gal-I is further increased in metastases [46] and that this increase is associated with poor prognosis [47]. Recent studies suggested that ST6Gal-I played an important role in regulating gene expression and protein glycosylation.

In the present study, we showed that the down-regulation of ST6Gal-I in cervical cancer is associated with decreased tumor cell proliferation, invasion and resistance to cisplatin. Several previous studies demonstrated similar effects of ST6Gal-I silencing in different types of cells, such as MDA-MB-435 breast carcinoma cell. Their results suggest that cell surface α2,6-sialylation contributes to cell-cell and cell-extracellular matrix adhesion of tumor cells. Inhibition of sialytransferase ST6Gal-I by antisense-oligodeoxynucleotides might be a way to reduce the metastatic capacity of carcinoma cells [20]. Based on the results of previous studies, we used the MTT assay to examine the effect of ST6Gal-I knockdown on the proliferation of HeLa cells in response to DDP treatment and we determined the optimal DDP concentration and treatment time for subsequent experiments. We used flow cytometry and western blotting to detect the expression of α-2,6-sialic acid in HeLa cells and confirmed the efficiency of shRNA-mediated ST6Gal-I silencing in these cells. Additionally, the role of ST6Gal-I in cisplatin-resistance in cervical cancer cells was investigated. ShRNA-mediated knockdown of ST6Gal-I in HeLa cells inhibited cell proliferation and increased cisplatin sensitivity. A previous study suggested that the role of ST6Gal-I in the regulation of cell proliferation and drug response is tumor type-specific. However, in the present study, the effect of ST6Gal-I silencing on invasion was more significant than that on cell survival and cisplatin resistance, suggesting that there are other factors besides ST6Gal-I affecting these pathways. On the other hand, it is important to evaluate both the toxicity and potency of ST6Gal-I silencing in chemotherapy in vivo. Patient-derived xenograft models could serve as a link between clinical research and in vitro studies in cell lines. Our present analysis showed that tumor volume in ST6Gal-I silenced BALB/c mice treated with DDP was significantly decreased, indicating that knockdown of ST6Gal-I enhanced the effect of cisplatin on the suppression of subcutaneous tumor growth. The immunohistochemical results of ST6Gal-I protein expression further validated the down-regulation effect of ST6Gal-I shRNA in xenograft tumor tissues.

Apoptosis or programmed cell death is an evolutionarily conserved cellular process that is required for normal embryonic development and maintenance of tissue homeostasis. The B-cell lymphoma protein-2 (BCL-2) family proteins are essential regulators of apoptosis [48]. Furthermore, BCL-2 is a known repressor of apoptosis, and a positive regulator of cell differentiation. Aberrant expression and function of BCL-2 family members results in de-regulation of apoptosis that contributes to the development of a variety of human pathologies including cancer [49]. The weaker protein expression of BCL-2 suggests the increased cell apoptosis. In the present study, detection of BCL-2 protein expression in xenograft tumor tissues showed that the expression of BCL-2 was weakest in ST6Gal-I-shRNA + DDP group, and thus the increased apoptosis in HeLa cells. The results suggested knockdown of ST6Gal-I increased cisplatin sensitivity in cervical cancer cells.

## Conclusions

In summary,the results of the present study showed that loss of ST6Gal-I promotes cell apoptosis, inhibits the invasive ability of cells and increases the sensitivity of cervical cancer cells to cisplatin. Further investigation into the coordinated effects of target modulation and anti-cancer drugs may promote the development of combination therapy with sialyltransferase inhibitors and cisplatin for the treatment of recurrent and metastatic cervical cancer.

## Additional file

Additional file 1: Figure S1. ST6Gal-I mRNA expression levels after transfection for different time points analyzed in this study. (TIF 21 kb)

## Abbreviations

ANOVA: Two-way analysis of variance
CMP: Cytidine monophosphate
Col: Collagen
DDP: Cisplatin
DMEM: Dulbecco's modified eagle's medium
ECM: Extracellular matrix
FITC: Fluorescein isothiocyanate
Fn: Fibronectin
HE: Hematoxylin-eosin
IARC: International agency for research on cancer
IC_50_: 50% inhibition rate
Ln: Laminin
MDR: Multidrug resistance
MTT: 3-(4,5-dimethylthiazol-2-yl)-2-5-diphenyl tetrazolium bromide
NAC: Neoadjuvant chemotherapy
Neu5Ac: N-acetylneuraminic acids
PBS: Phosphate-buffered saline
PI: Propidium iodide
SD: Standard deviation
shRNA: Short hairpin RNA
SPSS: Statistical package for the social sciences
ST6Gal-I: Sialyltransferase I

## References

[1] Lei C, Wang Y, Huang Y, Yu H, Huang Y, Wu L, Huang L (2012). Up-regulated miR155 reverses the epithelial-mesenchymal transition induced by EGF and increases chemo-sensitivity to cisplatin in human Caski cervical cancer cells. PLoS One.

[2] Ma D, Zhang YY, Guo YL, Li ZJ, Geng L (2012). Profiling of microRNA-mRNA reveals roles of microRNAs in cervical cancer. Chin Med J.

[3] Guo L, Zhu H, Lin C, Che J, Tian X, Han S, Zhao H, Zhu Y, Mao D (2015). Associations between antioxidant vitamins and the risk of invasive cervical cancer in Chinese women: A case-control study. Sci Rep.

[4] Shen Y, Yang L, Wang Z (2012). Treatment of early bulky cervical cancer with neoadjuvant paclitaxel, carboplatin and cisplatin prior to laparoscopical radical hysterectomy and pelvic lymphadenectomy. Oncol Lett.

[5] Benedetti-Panici P, Greggi S, Scambia G, Amoroso M, Salerno MG, Maneschi F, Cutillo G, Paratore MP, Scorpiglione N, Mancuso S (1998). Long-term survival following neoadjuvant chemotherapy and radical surgery in locally advanced cervical cancer. Eur J Cancer.

[6] Choi CH, Kim TJ, Lee JW, Kim BG, Lee JH, Bae DS (2007). Phase II study of neoadjuvant chemotherapy with mitomycin-c, vincristine and cisplatin (MVC) in patients with stages IB2-IIB cervical carcinoma. Gynecol Oncol.

[7] Arriagada R, Dunant A, Pignon JP, Bergman B, Chabowski M, Grunenwald D, Kozlowski M, Le Pechoux C, Pirker R, Pinel MI (2010). Long-term results of the international adjuvant lung cancer trial evaluating adjuvant Cisplatin-based chemotherapy in resected lung cancer. J Clin Oncol.

[8] Helbig L, Damrot J, Hulsenbeck J, Koberle B, Brozovic A, Osmak M, Fiket Z, Kaina B, Fritz G (2011). Late activation of stress-activated protein kinases/c-Jun N-terminal kinases triggered by cisplatin-induced DNA damage in repair-defective cells. J Biol Chem.

[9] Li K, Chen B, Xu L, Feng J, Xia G, Cheng J, Wang J, Gao F, Wang X (2013). Reversal of multidrug resistance by cisplatin-loaded magnetic Fe3O4 nanoparticles in A549/DDP lung cancer cells in vitro and in vivo. Int J Nanomedicine.

[10] Jiang Z, Chen BA, Xia GH, Wu Q, Zhang Y, Hong TY, Zhang W, Cheng J, Gao F, Liu LJ (2009). The reversal effect of magnetic Fe3O4 nanoparticles loaded with cisplatin on SKOV3/DDP ovarian carcinoma cells. Int J Nanomedicine.

[11] Shen Y, Ren M, Shi Y, Zhang Y, Cai Y (2011). Octreotide enhances the sensitivity of the SKOV3/DDP ovarian cancer cell line to cisplatin chemotherapy in vitro. Exp Ther Med.

[12] Joyce JA, Pollard JW (2009). Microenvironmental regulation of metastasis. Nat Rev Cancer.

[13] von Wichert G, Sheetz MP (2005). Mechanisms of disease: the biophysical interpretation of the ECM affects physiological and pathophysiological cellular behavior. Z Gastroenterol.

[14] Hynes RO (2002). Integrins: bidirectional, allosteric signaling machines. Cell.

[15] Yu S, Fan J, Liu L, Zhang L, Wang S, Zhang J (2013). Caveolin-1 up-regulates integrin alpha2,6-sialylation to promote integrin alpha5beta1-dependent hepatocarcinoma cell adhesion. FEBS Lett.

[16] Gu J, Isaji T, Sato Y, Kariya Y, Fukuda T (2009). Importance of N-glycosylation on alpha5beta1 integrin for its biological functions. Biol Pharm Bull.

[17] Wang PH, Lee WL, Lee YR, Juang CM, Chen YJ, Chao HT, Tsai YC, Yuan CC (2003). Enhanced expression of alpha 2,6-sialyltransferase ST6Gal I in cervical squamous cell carcinoma. Gynecol Oncol.

[18] Kaneko Y, Yamamoto H, Kersey DS, Colley KJ, Leestma JE, Moskal JR (1996). The expression of Gal beta 1,4GlcNAc alpha 2,6 sialyltransferase and alpha 2,6-linked sialoglycoconjugates in human brain tumors. Acta Neuropathol.

[19] Dall'Olio F, Chiricolo M, D'Errico A, Gruppioni E, Altimari A, Fiorentino M, Grigioni WF (2004). Expression of beta-galactoside alpha2,6 sialyltransferase and of alpha2,6-sialylated glycoconjugates in normal human liver, hepatocarcinoma, and cirrhosis. Glycobiology.

[20] Lin S, Kemmner W, Grigull S, Schlag PM (2002). Cell surface alpha 2,6 sialylation affects adhesion of breast carcinoma cells. Exp Cell Res.

[21] Nambudiri VE, Widlund HR (2013). Small interfering RNA. J Invest Dermatol.

[22] Antony P, Rose M, Heidenreich A, Knuchel R, Gaisa NT, Dahl E (2014). Epigenetic inactivation of ST6GAL1 in human bladder cancer. BMC Cancer.

[23] Bian HB, Pan X, Yang JS, Wang ZX, De W (2011). Upregulation of microRNA-451 increases cisplatin sensitivity of non-small cell lung cancer cell line (A549). J Exp Clin Cancer Res.

[24] Jing Z, Heng W, Xia L, Ning W, Yafei Q, Yao Z, Shulan Z (2015). Downregulation of phosphoglycerate dehydrogenase inhibits proliferation and enhances cisplatin sensitivity in cervical adenocarcinoma cells by regulating Bcl-2 and caspase-3. Cancer Biol Ther.

[25] Yang C, Cai J, Wang Q, Tang H, Cao J, Wu L, Wang Z (2012). Epigenetic silencing of miR-130b in ovarian cancer promotes the development of multidrug resistance by targeting colony-stimulating factor 1. Gynecol Oncol.

[26] Moremen KW, Tiemeyer M, Nairn AV (2012). Vertebrate protein glycosylation: diversity, synthesis and function. Nat Rev Mol Cell Biol.

[27] Zoldos V, Novokmet M, Beceheli I, Lauc G (2013). Genomics and epigenomics of the human glycome. Glycoconj J.

[28] Schachter H, Freeze HH (2009). Glycosylation diseases: quo vadis?. Biochim Biophys Acta.

[29] Stanley P, Schachter H, Taniguchi N. N-glycans. In: Varki A, Cummings RD, Esko JD, Freeze HH, Stanley P, Bertozzi CR, Hart GW, Etzler ME. Discovery and Classification of Glycan-Binding Proteins -- Essentials of Glycobiology. Cold Spring Harbor: Cold Spring Harbor Laboratory Press; 2009. p. 101–14. 20301249

[30] Brockhausen I, Schachter H, Stanley P. O-GalNAc glycans. In: Varki A, Cummings RD, Esko JD, Freeze HH, Stanley P, Bertozzi CR, Hart GW, Etzler ME. Chemical and Enzymatic Synthesis of Glycans and Glycoconjugates -- Essentials of Glycobiology. Cold Spring Harbor: Cold Spring Harbor Laboratory Press; 2009. p. 115–28.

[31] Miyagi T, Takahashi K, Hata K, Shiozaki K, Yamaguchi K (2012). Sialidase significance for cancer progression. Glycoconj J.

[32] Dall'Olio F, Malagolini N, Trinchera M, Chiricolo M (2012). Mechanisms of cancer-associated glycosylation changes. Front Biosci (Landmark Ed).

[33] Schultz MJ, Swindall AF, Bellis SL (2012). Regulation of the metastatic cell phenotype by sialylated glycans. Cancer Metastasis Rev.

[34] Adamczyk B, Tharmalingam T, Rudd PM (2012). Glycans as cancer biomarkers. Biochim Biophys Acta.

[35] Varki A (2008). Sialic acids in human health and disease. Trends Mol Med.

[36] Yamaguchi K, Shiozaki K, Moriya S, Koseki K, Wada T, Tateno H, Sato I, Asano M, Iwakura Y, Miyagi T (2012). Reduced susceptibility to colitis-associated colon carcinogenesis in mice lacking plasma membrane-associated sialidase. PLoS One.

[37] Varki NM, Varki A (2007). Diversity in cell surface sialic acid presentations: implications for biology and disease. Lab Invest.

[38] Varki A (2010). Colloquium paper: uniquely human evolution of sialic acid genetics and biology. Proc Natl Acad Sci U S A.

[39] Li Y, Chen X (2012). Sialic acid metabolism and sialyltransferases: natural functions and applications. Appl Microbiol Biotechnol.

[40] Audry M, Jeanneau C, Imberty A, Harduin-Lepers A, Delannoy P, Breton C (2011). Current trends in the structure-activity relationships of sialyltransferases. Glycobiology.

[41] Chen X, Varki A (2010). Advances in the biology and chemistry of sialic acids. ACS Chem Biol.

[42] Tsuji S (1996). Molecular cloning and functional analysis of sialyltransferases. J Biochem.

[43] Harduin-Lepers A, Recchi MA, Delannoy P (1995). 1994, the year of sialyltransferases. Glycobiology.

[44] Datta AK (2009). Comparative sequence analysis in the sialyltransferase protein family: analysis of motifs. Curr Drug Targets.

[45] Harduin-Lepers A, Vallejo-Ruiz V, Krzewinski-Recchi MA, Samyn-Petit B, Julien S, Delannoy P (2001). The human sialyltransferase family. Biochimie.

[46] Costa-Nogueira C, Villar-Portela S, Cuevas E, Gil-Martin E, Fernandez-Briera A (2009). Synthesis and expression of CDw75 antigen in human colorectal cancer. BMC Cancer.

[47] Lise M, Belluco C, Perera SP, Patel R, Thomas P, Ganguly A (2000). Clinical correlations of alpha2,6-sialyltransferase expression in colorectal cancer patients. Hybridoma.

[48] Phillips DC, Xiao Y, Lam LT, Litvinovich E, Roberts-Rapp L, Souers AJ, Leverson JD (2015). Loss in MCL-1 function sensitizes non-Hodgkin's lymphoma cell lines to the BCL-2-selective inhibitor venetoclax (ABT-199). Blood Cancer J.

[49] Youle RJ, Strasser A (2008). The BCL-2 protein family: opposing activities that mediate cell death. Nat Rev Mol Cell Biol.

